# Mesenchymal and induced pluripotent stem cell–based therapeutics: a comparison

**DOI:** 10.1007/s00253-023-12583-4

**Published:** 2023-05-29

**Authors:** Misha A. Teale, Samuel Schneider, Dieter Eibl, Christian van den Bos, Peter Neubauer, Regine Eibl

**Affiliations:** 1 Centre for Biochemical Engineering and Cell Cultivation Techniques, Institute of Chemistry and Biotechnology, Zurich University of Applied Sciences, Grüentalstrasse 14, 8820 Wädenswil, Switzerland; 2 Mares Advanced Therapies, 48268 Greven, Germany; 3 Institute of Biotechnology, Chair of Bioprocess Engineering, Technical University of Berlin, ACK24, Ackerstraße 76, 13355 Berlin, Germany

**Keywords:** Allogeneic, Safety, Single-use systems, Scale-up, Upstream processing, Downstream processing

## Abstract

**Abstract:**

Stem cell–based cell therapeutics and especially those based on human mesenchymal stem cells (hMSCs) and induced pluripotent stem cells (hiPSCs) are said to have enormous developmental potential in the coming years. Their applications range from the treatment of orthopedic disorders and cardiovascular diseases to autoimmune diseases and even cancer. However, while more than 27 hMSC-derived therapeutics are currently commercially available, hiPSC-based therapeutics have yet to complete the regulatory approval process. Based on a review of the current commercially available hMSC-derived therapeutic products and upcoming hiPSC-derived products in phase 2 and 3, this paper compares the cell therapy manufacturing process between these two cell types. Moreover, the similarities as well as differences are highlighted and the resulting impact on the production process discussed. Here, emphasis is placed on (i) hMSC and hiPSC characteristics, safety, and ethical aspects, (ii) their morphology and process requirements, as well as (iii) their 2- and 3-dimensional cultivations in dependence of the applied culture medium and process mode. In doing so, also downstream processing aspects are covered and the role of single-use technology is discussed.

**Key points:**

*• Mesenchymal and induced pluripotent stem cells exhibit distinct behaviors during cultivation*

*• Single-use stirred bioreactor systems are preferred for the cultivation of both cell types*

*• Future research should adapt and modify downstream processes to available single-use devices*

## Introduction

The application of stem cells in medicine is by no means a modern idea, and with the cell therapy market size expected to grow to $45 billion by 2030 (Vision Research Reports 2022) due to strong financial backing and lower regulatory hurdles, especially in North America (Polaris Market Research 2022), a closer look at the cells driving this trend is definitely warranted. General interest in the regenerative properties of stem cells first began as far back as 1867, when Cohnheim (1867) observed how non-hematopoietic cells migrated to the site of inflammation and differentiated to fibroblasts during wound healing. A century and a half of research finally led to the identification (Rekers 1950) and general characterization (Tavassoli and Crosby 1968; Friedenstein et al. 1970; Owen and Friedenstein 1988; Pittenger et al. 1999; van den Bos et al. 2014) of hMSCs. These cells not only have the ability to replace damaged tissue via differentiation, but also produce and secrete chemo- and cytokines, modulating local immune response and tissue regeneration (van den Bos et al. 2014; Zhou et al. 2019). In this context, hMSC-derived extracellular vesicles have also been the target of recent cell-free therapeutics. A more comprehensive review of these products may be found in the publication by Kou et al. (2022).

Currently, more than 1400 clinical trials have been submitted involving the application of hMSCs as regenerative medicine products or biologics in the USA alone (National Library of Medicine (US) 2022), and more than 27 products containing hMSCs have been approved in several countries worldwide (Table 1). Of these 27 products, 17 are allogeneic, meaning the cells from a single healthy donor are used to treat multiple patients, while only 10 are autologous, i.e., the donor is the patient, displaying a clear trend towards allogenic. The first such product to be granted orphan drug status by the European Union and to receive market approval in 2009 for the treatment of perianal fistulas resulting from Crohn’s disease or inflammatory bowel disease was Alofisel (European Medicines Agency 2009). Since then, many more have followed, e.g., for the treatment of osteoarthritis (Ha et al. 2019), graft-versus-host disease (Heathman et al. 2015), and spinal cord injury (Cofano et al. 2019).

**Table 1:** Overview of approved hMSC-based therapeutics and hiPSC-based therapeutics in phase 2 and 3 clinical trials

Company	Medicinal product	Cell type	Indication	Phase	Marketing authorization
AlloSource	AlloStem^®^	Allogeneic hAD-MSCs	Bone regeneration	Approved	USA
Anterogen Co., Ltd.	Cupistem^®^	Autologous hAD-MSCs	Crohn’s fistula	Approved	South Korea
	Adipocell^®^	Autologous hAD-MSCs	Chronic ischemic cardiomyopathy	Approved	South Korea
	Queencell^®^	Autologous hAD-MSCs	Subcutaneous tissue defect	Approved	South Korea
Astellas Pharma	MA09-hRPE	Allogeneic hiPSC-RPEs	Macular degeneration	Phase 1 | phase 2	n.a.
Biomet Inc.	Bonus Triad™	Allogeneic hBM-MSCs	Musculoskeletal defects	Approved	USA
Cell Tech Pharmed Company	Mesetrocell^®^	Autologous hBM-MSCs	Multiple sclerosis	Approved	Iran
Corestem, Inc.	NeuroNata-R^®^	Autologous hBM-MSCs	Amyotrophic lateral sclerosis	Approved	South Korea
Cynata Therapeutics Ltd.	CYP-004	Allogeneic hiPSC-MSCs	Osteoarthritis	Phase 3	n.a.
	CYP-001	Allogeneic hiPSC-MSCs	Graft-versus-host disease	Phase 2	n.a.
	CYP-002	Allogeneic hiPSC-MSCs	Critical limb ischemia	Phase 2	n.a.
	CYP-001	Allogeneic hiPSC-MSCs	Acute respiratory distress syndrome	Phase 1 | phase 2	n.a.
JCR Pharmaceuticals	TEMCELL^®^ HS	Allogeneic hBM-MSCs	Graft-versus-host disease	Approved	Japan
medac GmbH	Obnitix®	Allogeneic hBM-MSCs	Graft-versus-host disease	Approved	Germany
Medipost Co. Ltd.	CARTISTEM^®^	Allogeneic hUC-MSCs	Osteoarthritis	Approved	South Korea
Mesoblast, Inc.	Remestemcel-L	Allogeneic hBM-MSCs	Graft-versus-host disease	Approved	Canada/New Zealand
Nipro Corporation	Stemirac^®^	Autologous hBM-MSCs	Spinal cord injury	Approved	Japan
NuVasive	OsteoCel Plus	Allogeneic hBM-MSCs	Spinal cord injury	Approved	USA
Orthofix Inc.	Trinity Evolution™	Allogeneic hBM-MSCs	Musculoskeletal defects	Approved	USA
	Trinity Elite™	Allogeneic hBM-MSCs	Musculoskeletal defects	Approved	USA
Osiris Therapeutics, Inc.	BIO4^®^	Allogeneic hBM-MSCs	Musculoskeletal defects	Approved	USA
Pharmicell Co., Ltd.	Cellgram^®^	Autologous hBM-MSCs	Acute myocardial infarction	Approved	South Korea
Regeneus Ltd.	HiQCell^®^	Autologous hAD-MSCs	Musculoskeletal defects	Approved	Australia
Reliance Life Sciences	CardioRel^®^	Autologous hBM-MSCs	Myocardial infarction	Approved	India
ReNeuron Ltd.	CTX0E03	Allogeneic hiPSC-NSCs	Ischemic stroke	Phase 2	n.a.
	hRPC	Allogeneic hiPSC-RPCs	Retinitis pigmentosa	Phase 1 | phase 2	n.a.
Sewon Cellontech Co., Ltd.	RMS Ossron™	Autologous hBM-MSCs	Bone regeneration	Approved	South Korea
Smith & Nephew	Grafix^®^	Allogeneic hUC-MSCs	Advanced wound therapy	Approved	USA
	GrafixPL^®^	Allogeneic hUC-MSCs	Advanced wound therapy	Approved	USA
	Stravix^®^	Allogeneic hUC-MSCs	Diabetic wound	Approved	USA
	StravixPL^®^	Allogeneic hUC-MSCs	Diabetic wound	Approved	USA
Stempeutics Research Pvt Ltd	Stempeucel^®^	Allogeneic hBM-MSCs	Critical limb ischemia	Approved	India
	Stempeucel^®^	Allogeneic hBM-MSCs	Osteoarthritis	Filed	India
Takeda	Alofisel^®^	Allogeneic hAD-MSCs	Crohn’s fistula	Approved	EU/Japan

*hAD-MSCs* human adipose tissue–derived mesenchymal stem cells (hAD-MSCs), *hBM-MSCs* human bone marrow–derived mesenchymal stem cells, *hUC-MSCs* human umbilical cord–derived mesenchymal stem cells, *hiPSC-RPEs* human-induced pluripotent-derived retinal pigment epithelial cells, *hiPSC-MSCs* human-induced pluripotent-derived mesenchymal stem cells, *hiPSC-NSCs* human-induced pluripotent-derived neural stem cells, *hiPSC-RPCs* human-induced pluripotent-derived retinal pigment cells, *n.a.* not applicable

Compared to hMSCs, hiPSCs are a far more recent discovery (Takahashi and Yamanaka 2006; Takahashi et al. 2007a; Flahou et al. 2021). These cells are characterized by their inherent capacity for indefinite self-renewal and ability to differentiate into all three germ layers (endo-, meso-, and ectoderms), which allows a broader range of indications to be targeted than would be possible with hMSCs. To this end, their suitability for the treatment of diseases currently considered challenging using conventional means, such as macular degeneration, ischemic stroke, and cancer (Shiba et al. 2016; Takagi et al. 2019), has been repeatedly demonstrated. In fact, more than 27 hiPSC-derived cell products are currently undergoing pre-clinical to phase 3 trials (National Library of Medicine (US) 2022), 7 of which are in phases 2 and 3 (Table 1). Of these 7 products, all are based on the allogeneic manufacturing approach. Companies currently leading the development of such hiPSC-based cell therapeutics include Astellas Pharma, Cynata Therapeutics Ltd., and ReNeuron Ltd.

Regardless of whether the cell therapy is based on hMSCs or hiPSCs, it stringently requires the production of clinically relevant cell quantities of between 10^5^ and 10^12^ per dose while ensuring target cell quality (viability, marker profile, potency), both of which have been linked to therapeutic efficacy and are outlined in more detail elsewhere (Dominici et al. 2006; Sullivan et al. 2018; Scibona and Morbidelli 2019). This review therefore focuses on the upstream and downstream processing for both hMSC and hiPSC-based cell therapeutics. Furthermore, it highlights successfully implemented single-use (SU) devices, while considering cell typical characteristics and requirements.

## hMSCs versus hiPSCs

### Origin, safety, and ethical aspects

The production process of hMSC- and hiPSC-based therapeutics always begins with the extraction of tissue from a willing donor (see also Fig. 2). For example, hMSCs, which belong to the group of multipotent adult stem cells, are easily accessible and present in almost every human organ (Audet and Stanford 2009). To date, no safety concerns have been reported regarding their use, likely due to their immune-privileged status (Najar et al. 2022). In addition, their use is not ethically objectionable, as they are obtained from consenting individuals (Cofano et al. 2019). Historically, the most important source of hMSCs has been bone marrow (Audet and Stanford 2009). However, larger quantities of hMSCs can easily be obtained from adipose tissue as a byproduct of liposuction (Timaner et al. 2020) or from the umbilical cord immediately after birth (Audet and Stanford 2009). Consequently, the umbilical cord–derived subtype has also exhibited a superior life span, lower risk of contamination, and better immunological compatibility compared to the bone marrow–derived subtype (Mahmood et al. 2018).

In contrast to hMSCs, hiPSCs are pluripotent stem cells produced by reprogramming (Takahashi et al. 2007a; Hsu et al. 2018) of somatic skin (Takahashi et al. 2007b; Yu et al. 2007) or blood cells (Zeng et al. 2017) in the laboratory. This reprogramming was initially realized through the introduction of 4 factors (Oct3/4, Sox2, Kfl4, and c-Myc) via a viral vector transport vehicle which integrated them into the host cell DNA. The forced expression of these factors returned the cells to an embryonic state, allowing them to once again differentiate into all germ layers (Takahashi et al. 2007a). In this manner, the ethical issues of using promising embryonic stem cell–derived therapeutic products (Menasché 2020) were circumvented, earning Shinya Yamanaka, the researcher who pioneered the method with murine cells in 2006 (Takahashi and Yamanaka 2006), the Nobel Prize in 2012 (The Nobel Prize 2022).

Although the generation of hiPSCs is ethically acceptable and technically simple, it still remains inefficient, time-consuming, and expensive (Borgohain et al. 2019). Currently, it takes several months until sufficient cells are available for experiments. Also, reprogramming efficiencies reported in the literature are generally between 0.001 and 1%, meaning that at most, only 1 out of 100 cells is successfully reprogrammed into a hiPSC (Birbriar 2021).

Another important consideration is safety. The integration of the previously mentioned factors into the genome of hiPSCs during reprogramming increases the risk of interference with other important genes, which may lead to tumor formation in vivo following implantation (Yu et al. 2007), even after directed differentiation (Lee et al. 2013; Kojima et al. 2019). Other risks include the occurrence of genetic abnormalities during ex vivo cultivation and tissue rejection by the patient’s immune system, neither of which can be completely precluded at present (Sullivan et al. 2018; Rehakova et al. 2020). Consequently, hiPSCs are considered less safe than hMSCs, which also explains the lack of commercially available hiPSC-based therapeutics to date. Regardless, researchers are working expeditiously towards developing more safe and efficient reprogramming techniques (Rajasingh et al. 2021). These include the use of more responsive and accessible tissues (Rajasingh et al. 2021), more potent and lower risk gene combinations for reprogramming (Okita et al. 2007; Yu et al. 2007; Furukawa et al. 2022), and the use of non-integrating vectors or even completely non-viral methods (Birbriar 2021).

### Cell characteristics and impact on cultivation conditions

Once a cell line has been established, a closer look at its characteristics must be taken to ensure optimal cell growth. In this regard, both hMSCs and hiPSCs require a temperature of 37 °C and a pH between 6.9 and 7.5. In addition, both cell types can be cultivated under normoxic as well as hypoxic conditions (Antebi et al. 2018), are considered shear sensitive (Horiguchi et al. 2021; Burns et al. 2021), and are strictly adherent. The latter characteristic describes their need for a planar surface or scaffold to survive and self-renew. These cells are therefore either cultivated in static cultivation systems as a monolayer (2D culture), or in mechanically or hydraulically driven dynamic bioreactors (3D culture), as spheroids (cell aggregates), or on artificial scaffolds, such as membranes, macrocarriers or microcarriers.

**Fig. 1:**
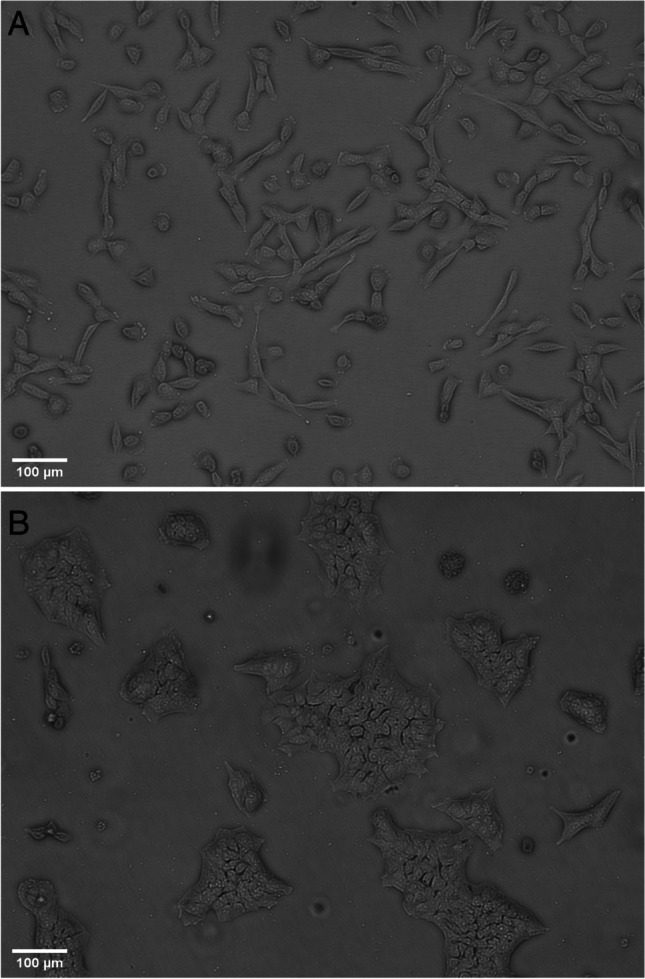
Phase contrast images taken of the ATCC^®^ adipose-derived mesenchymal stem cell line SCRC-4000™ (**A**) and the Gibco™ episomal induced pluripotent stem cell line (**B**) cultivated as monolayers on coated polystyrene surfaces. The scale bar in the lower left corner corresponds to 100 μm

In both 2D and 3D cultivations, differences in size, morphology, and motility impact spatial requirements and must therefore be considered. For example, in suspension, single hMSCs and hiPSCs are roughly spherical with a similar diameter of approximately 18 μm (Pittenger et al. 2019) and 16 μm (Lipsitz et al. 2018), respectively, and are able to form and proliferate as spheroids (Allen et al. 2019). However, after attachment to an artificial scaffold, predominantly via integrin (Jin et al. 2012; Isomursu et al. 2019), their morphology and size differ significantly (Fig. 1). While hMSCs adopt a fibroblast-like morphology and require between 450 and 35,000 μm^2^ per cell (Haasters et al. 2009), hiPSCs only require ≈43.5 μm^2^ due to their epithelial morphology (Wakao et al. 2012; Courtot et al. 2014). Hence, more hiPSCs can be grown per available surface area before confluency is reached. They also display a high nucleus-to-cytoplasm ratio (Wakao et al. 2012), a characteristic associated with proliferative potential (Wang et al. 2021).

The effective use of limited spatial resources can further be optimized through single-cell passaging and by ensuring uniform cell distribution during inoculation. In this regard, hMSCs are less susceptible as they display higher motility (Bertolo et al. 2015; Somaiah et al. 2015) and are able to migrate to sites with lower relative cell occupation, delaying the onset of localized confluency and contact inhibition. In contrast, hiPSCs remain in very close proximity to their point of initial attachment (Zhang et al. 2011) and are therefore more susceptible to inhomogeneous inoculation. Regardless, the migratory capacity of hMSCs has been shown to decrease and their size increase, as replicative senescence sets in (Haasters et al. 2009; Bertolo et al. 2015), potentially impacting this phenomenon alongside overall cell quality. This is currently estimated to happen after approximately 20–40 population doublings (Khorraminejad-Shirazi et al. 2019). Assuming one can isolate 10^3^–10^4^ cells (Stocchero and Stocchero 2011), 16–20 population doublings would be required to produce 1 dose containing 10^9^ cells with the target cell quality (Scibona and Morbidelli 2019). Practically, cell cultivation and purification results in cell loss and far lower yields must be expected. Consequently, the manner and duration of these steps have a significant impact on cell yield and quality per batch, following cultivation, harvest, purification, and cryogenic storage, which has prompted the development of bioprocessing solutions to meet these needs.

## The production of hMSC- and hiPSC-based therapeutics

### Manufacturing overview

**Fig. 2:**
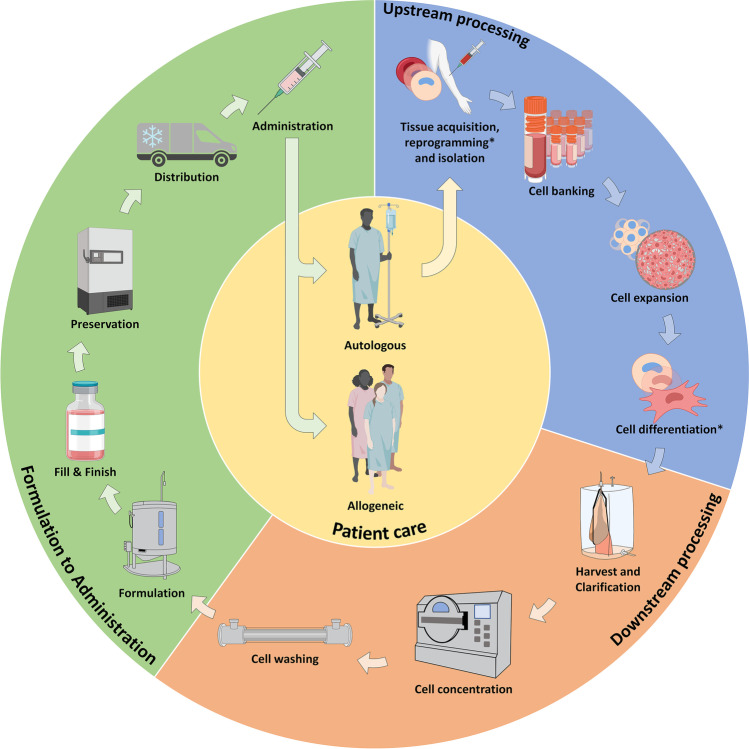
A simplified workflow for the production of hMSC-based and hiPSC-based therapeutics. The manufacturing process has been divided into (blue) typical upstream and (orange) downstream operations, followed by (green) the formulation to administration steps. Process steps marked with an asterisks only concern hiPSC-based products (created with Biorender.com)

Typical bioprocessing steps for hMSC- and hiPSC-based therapeutics are outlined in Fig. 2. The manufacturing of these cells begins with the procedures up to and including the cellular expansion or differentiation stage, referred to as upstream processing. While hMSCs only undergo expansion during the upstream process, hiPSCs must also undergo differentiation into the desired cell types, such as cardiomyocytes, insulin-producing pancreatic cells, or neurons, for safety reasons (Kojima et al. 2019). Here cultivation conditions, such as medium composition and shear stress, may be used to realize the differentiation process (Yourek et al. 2010; Gultian et al. 2022). Following the upstream process, downstream processing generally involves cell harvest, clarification, concentration, and washing (Cunha et al. 2015a; Jossen et al. 2018). Thereafter, the cell suspension undergoes formulation, final fill and finish, cryogenic storage, distribution, and patient administration.

### Upstream processing

#### Bioreactors and process mode

To maintain target cell quality and growth throughout the entire upstream process, careful attention must be paid to maintaining a well-defined environment with consistent mechanical, physical, and chemical cues (García-Fernández et al. 2020). According to literature, this is best ensured through either mechanical or hydraulic agitation (Jossen et al. 2018; Tsai and Pacak 2021) in instrumented bioreactors (see also Table 2), which allow for the regulation and near homogenous distribution of process parameters such as temperature, pH, and dissolved oxygen (Manstein et al. 2021). Our review further revealed that most hMSC and hiPSC cultivations were performed in top-driven stirred bioreactor systems using microcarriers (Schirmaier et al. 2014; Dufey et al. 2016; Lawson et al. 2017; Pandey et al. 2020; Rotondi et al. 2021). A more detailed overview of microcarrier types suitable for stem cell production is provided in a review by Ornelas-González et al. (2021). Furthermore, while hMSC cultivation up to a pilot scale of 150 L has been described using stirred bioreactors (Jossen et al. 2018), hiPSC expansion has not yet progressed beyond bench-top scale (Pandey et al. 2020).

**Table 2:** Instrumented SU bioreactors used for the expansion of hMSCs and hiPSCs. Listed are approximate maximum cell yields per cultivation, the expansion factor achieved and cultivation duration. The expansion factor was calculated as the ratio between the maximum number of cells reached and the number used for inoculation

Working principle	Working volume (range)	Bioreactor	Vendor	Cells	Cultivation mode	Cultivation type	Max. cell yield [10^6^ cells]	Expansion factor (Day)	Reference
Mechanically driven: Stirred tank	15 mL–0.25 L	ambr^®^ 15	Sartorius AG	hBM-MSCs	Batch	Microcarriers	13*	44 (7)*	Rafiq et al. (2017)
		ambr^®^ 250	Sartorius AG	hMSCs	Repeated batch	Microcarriers	37*	6.1 (11)*	Rotondi et al. (2021)
		BioBLU^®^ 0.3c SU	Eppendorf AG	hiPSCs	Perfusion	Spheroids	713	5.7 (7)	Kropp et al. (2016)
				hBM-MSCs	Repeated batch	Microcarriers	84	14 (15)	Dufey et al. (2016)
		Vertical-Wheel™	PBS Biotech	hiPSCs	Repeated batch	Spheroids	70*	35 (6)*	Lee et al. (2020)
				hBM-MSCs	Repeated batch	Microcarriers	53.1*	6.4 (7)*	de Almeida Fuzeta et al. (2020)
	0.4–8 L	BioBLU^®^ 3c SU	Eppendorf AG	hiPSCs	Perfusion	Microcarriers	5640	62.6 (9)	Pandey et al. (2020)
		BioBLU^®^ 5c SU	Eppendorf AG	hAD-MSCs	Repeated batch	Microcarriers	900	13.7 (16)	Siddiquee and Sha 2014)
		Mobius^®^ 3L SU	Merck Millipore	hiPSCs	Repeated batch	Spheroids	5970	10 (7)	Kwok et al. (2018)
				hAD-MSCs	Fed-batch	Microcarriers	670	46.5 (10)	Lawson et al. (2017)
		UniVessel SU 2L	Sartorius AG	hBM-MSCs	Repeated batch	Microcarriers	680	14 (7)	Cunha et al. (2017)
				hAD-MSCs			820	16 (7)	Cunha et al. (2017)
				hAD-MSCs	Repeated batch		540	35.4 (7)	Schirmaier et al. (2014)
		Vertical-Wheel™	PBS Biotech	hiPSCs	Repeated batch	Spheroids	472*	47.2 (6)*	Dang et al. (2021)
				hBM-MSCs		Microcarriers	660	12 (14)	Sousa et al. (2015)
		Xcellerex™ XDR-10	Cytiva	hiPSCs	Perfusion	Spheroids	10,200	51 (6)*	Huang et al. (2020)
	35–50 L	Allegro™ STR	Pall	hBM-MSCs	Fed-batch	Microcarriers	37,500	25 (6)	Pall Biotech (2020)
		BIOSTAT^®^ 50L STR	Sartorius AG	hAD-MSCs	Repeated batch	Microcarriers	10,850	97 (8)	Schirmaier et al. (2014)
		Mobius^®^ 50L	Merck Millipore	hAD-MSCs	Fed-batch	Microcarriers	12,800	42.7 (11)	Lawson et al. (2017)
Mechanically driven: Wave-mixed	0.5–2.4 L	BIOSTAT^®^ RM	Sartorius AG	hAD-MSCs	Repeated batch	Microcarriers	285	6.59 (9)	Jossen et al. (2016)
		WAVE	GE Healthcare	hPD-MSCs	Not specified	Microcarriers	n.i.	16.3 (7)	Timmins et al. (2012)
Mechanically driven: Multiplate	5.6 L	Xpansion^®^ 50	Pall	hPD-MSCs	Batch	Adherent	526.1	3.3 (7)	Lambrechts et al. (2016)
Hydraulically driven: Hollow fiber	n.a.	Quantum^®^ Cell Expansion System	Terumo BCT, Inc.	hiPSCs	Perfusion	Membrane	690	14 (6.5)	Paccola Mesquita et al. (2019)
				hAD-MSCs			605	28.8 (6)	Haack-Sørensen et al. (2018)
				hBM-MSCs			131	17.5 (13)	Mennan et al. (2019)
				hUC-MSCs			168	33.6 (8)	Mennan et al. (2019)
							483	24.2 (7)	Vymetalova et al. (2020)
Hydraulically driven: Fixed bed	0.5–3.5 L	Ascent	Corning	hAD-MSCs	Not specified	Adherent	161	6.42 (4)	Kiesslich (2022)

*hAD-MSCs* human adipose tissue–derived mesenchymal stem cells, *hBM-MSCs* human bone marrow–derived mesenchymal stem cells, *hiPSCs* human-induced pluripotent stem cells, *hMSCs* human mesenchymal stem cells, *hPD-MSCs* human placenta–derived mesenchymal stem cells, *hUC-MSCs* human umbilical cord–derived mesenchymal stem cells, *n.a.* not applicable

*Values approximated from publication figures

Independent of scale, one prevailing theme is the use of SU bioreactors for growing hMSCs (Schirmaier et al. 2014), hiPSCs (Kwok et al. 2018), and their differentiated progeny (Jiang et al. 2019; Shafa et al. 2019). Such bioreactors, whose sterile plastic containers are used only once, are currently offered by multiple vendors (Eibl and Eibl 2019; Eibl et al. 2022) and are available up to a maximum working volume of 6 m^3^. SU bioreactors are known to reduce the risk of cross-contamination (Jossen et al. 2018) and are approved and even recommended by regulatory agencies for production processes that are subject to stringent safety requirements, i.e., processes whereafter contaminants cannot be easily removed, such as stem cell expansion and differentiation for therapeutic use (Nogueira et al. 2021).

As shown in Table 2, the application of such SU bioreactors has enabled peak cell yields of between 10.9 and 37.5 × 10^9^ hMSCs or 10.2 × 10^9^ hiPSCs per batch, while still maintaining key cell quality indicators (Schirmaier et al. 2014; Lawson et al. 2017; Pall Biotech 2020; Huang et al. 2020). Our review has also shown that higher volumetric yields of hiPSCs can be expected per batch compared to hMSCs when using the same system. Another method of improving yield or reducing process time for both cell types was the choice of process mode. Here, the cultivations that achieved the highest hMSC and hiPSC expansion factors were performed either as repeated batch (Kwok et al. 2018; Pandey et al. 2020; Dang et al. 2021) or in perfusion mode (Abecasis et al. 2017; Pandey et al. 2020; Huang et al. 2020; Manstein et al. 2021). For perfusion processes with hMSCs and hiPSCs in stirred bioreactors, filters with either defined pore sizes (dos Santos et al. 2014; Kropp et al. 2016; Abecasis et al. 2017; Huang et al. 2020; Huang et al. 2020; Manstein et al. 2021), settling tubes (Huang et al. 2020; Sion et al. 2021), or acoustic separators (Huang et al. 2020) were used to ensure cell retention during medium replacement. Perfusion rates used for hiPSCs typically ranged from 0.5 to 1.3 vvd (Kropp et al. 2016; Abecasis et al. 2017; Pandey et al. 2020; Huang et al. 2020; Manstein et al. 2021), while for hMSCs, rates of 0.25 to 0.48 vvd (dos Santos et al. 2014; Sion et al. 2021) were used. In this manner, expansion factors of up to 33.6 and 62.6 in 8–9 days could be achieved for hMSCs (Mennan et al. 2019) and hiPSCs (Pandey et al. 2020), respectively.

While the *N*_*js*_ or *N*_*s*1_ and *N*_*s*1*u*_ criteria have been applied for stirred SU bioreactors operated with microcarriers to support the expansion of hMSCs and their process scale-up (Schirmaier et al. 2014; Lawson et al. 2017), other criteria, such as critical Kolmogorov length (*λ*_*c*_), have also been successfully used for the cultivation of hiPSCs as spheroids in both rotating- (Shafa et al. 2019) and vertical wheel-impeller (Dang et al. 2021) bioreactors. Based on Zwieterings (1958) findings, *N*_*js*_ or *N*_*s*1_ describes a minimum impeller speed at which solid particles, or in this case microcarriers, are just suspended in a bioreactor’s working volume (Hewitt et al. 2011; Rafiq et al. 2013), while *N*_*s*1*u*_ describes a lower limit for *N*_*js*_, where the microcarriers are in contact with the bottom of the bioreactor but not at rest (Kaiser et al. 2013; Jossen et al. 2016). These criteria may be determined visually or using particle image velocimetry (Tsai and Pacak 2021). Application of the *N*_*s*1*u*_ approach when cultivating hMSCs in spinner flasks allowed Jossen et al. (2016) to maintain a mean shear stress ($\overline{\tau}$) of 4.96 × 10^−3^ N m^−2^ ensuring optimal cell growth and quality. Petry and Salzig (2021) went on to define current estimates for acceptable $\overline{\tau}$ and energy dissipation rates ($\overline{\epsilon}$) in stirred bioreactors to be between 0.01–0.06 N m^−2^ and 0.2–4.8 mW kg^−1^, respectively, for hMSC cultivation. Additionally, they mentioned that the ratio of maximum energy dissipation (*ϵ*_*max*_) to $\overline{\epsilon}$, or hydrodynamic heterogeneity (*Φ*) should not exceed 20. While little has been reported on acceptable $\overline{\tau}$ ranges for hiPSCs, a $\overline{\epsilon}$ of 0.3–1.5 mW kg^−1^ has been suggested (Dang et al. 2021).

These authors also mention the use of *λ*_*c*_ as a means of estimating the maximum allowable power input in order to control spheroid size and minimize cell damage (Dang et al. 2021; Petry and Salzig 2021). Accordingly, the higher the impeller speed in a bioreactor, the smaller *λ*_*c*_ becomes, with its length ideally being more than two thirds of the diameter of any cell or microcarrier aggregates in the system, to prevent cell stripping at their liquid/aggregate interface (Hewitt et al. 2011; Nienow et al. 2016a; Nienow et al. 2016b). The direct application of *λ*_*c*_ does, however, presume fully turbulent conditions (Nienow 2021), which is generally not the case for the cultivation hMSCs or hiPSCs. Regardless, the approach has been readily adopted to limit the size of hMSC and hiPSC spheroids to a critical diameter of 200–300 μm (Sart et al. 2013; Allen et al. 2019; Huang et al. 2020; Petry and Salzig 2021), so that mass transfer is not restricted. These diameter limitations are less critical for microcarrier processes, especially when large spherical non-porous varieties (diameter ≈190 μm) are used, as they form more open aggregate structures (Ornelas-González et al. 2021). In this context, the ability of both cell types to detach from populated and reattach to unpopulated microcarriers or form bridges between the two, referred to as bead-to-bead transfer (Badenes et al. 2017; Leber et al. 2017; Rafiq et al. 2018), has also been observed. The use of bead-to-bead transfer during inoculation has been shown to reduce the lag phase associated with the proteolytic treatment of both cell types during single cell transfer from static to dynamic cultivation systems (Badenes et al. 2017; Rafiq et al. 2018), shortening process time.

#### Culture medium

In addition to the choice of bioreactor, process mode, and surface/scaffold, the culture medium has a decisive influence on the result of cell expansion and differentiation. Table 3 gives an overview of commercially available serum-free, xeno-free, or chemically defined media used for the production of hMSCs and hiPSCs. At this point, it is worth mentioning that while chemically defined media for cell differentiation are available (Gultian et al. 2022), their application remains limited. On the contrary, the more popular approach is to supplement the media either with fetal bovine serum or a substitute, such as KnockOut™ Serum Replacement (Ackermann et al. 2018), human plasma (Sivalingam et al. 2021), or platelet lysate (Mizukami et al. 2018) alongside other recombinant and synthetic components (Olmer et al. 2018; Haack-Sørensen et al. 2018; Yabe et al. 2019; Laco et al. 2020; Jacobson et al. 2021). Correspondingly, these media compositions have facilitated the differentiation of hiPSCs into various cell types, such as hMSCs (Goetzke et al. 2019), cardiomyocytes (Laco et al. 2020), neurons (Silva et al. 2021), definitive endoderm (Jacobson et al. 2021), and hematopoietic cells (Sivalingam et al. 2021). Moreover, by adjusting composition and leveraging changes in intrinsic metabolic requirements during differentiation, selective pressure could be applied, improving target cell purity (Kehoe et al. 2010; Tohyama et al. 2017; Hsu et al. 2021) prior to downstream processing.

**Table 3:** Commercially available chemically defined (CD) and xeno-free (XF) media used for the expansion of hMSCs and hiPSCs

Name	Manufacturer/developer	Cell type	Reference
StemFit^®^ (CD)	Amsbio LLC	hiPSCs	Morizane and Bonventre (2017)
StemXVivo XF Human MSC Expansion Medium	Bio-Techne AG	hMSCs	Bhat et al. (2021)
UrSuppe (CD)	Cardio Centero Ticino	hMSCs	Panella et al. (2021)
hiPSC Growth Medium (CD)	Cell Applications, Inc.	hiPSCs	Cell Applications, Inc. (2023a)
hMSC XF Basal Medium	Cell Applications, Inc.	hMSCs	Cell Applications, Inc. (2023b)
StemMaxOne (XF)	Cell Culture Technologies	hMSCs	Leber et al. (2017)
L7™ TFO2 (XF)	Lonza AG	hiPSCs	Pandey et al. (2020)
TheraPEAK™ MSCGM™ Mesenchymal Stem Cell Growth Medium (XF)	Lonza AG	hMSCs	Gottipamula et al. (2013)
Human Mesenchymal-XF Expansion Medium	Merck	hMSCs	Tang et al. (2022)
Stemline^®^ XF MSC Medium	Merck	hMSCs	Merck KGaA (2020)
StemMACS™ iPS-Brew XF	Miltenyi Biotec	hiPSCs	Lorenz et al. (2017)
PowerStem MSC1 (XF)	PAN-Biotech	hMSCs	Hoang et al. (2021)
Mesenchymal Stem Cell Growth Medium XF	PromoCell	hMSCs	Shetty et al. (2016)
NutriStem^®^ hPSC XF Medium	ReproCELL Inc.	hiPSCs	Jeriha et al. (2022)
RoosterNourish™-MSC-XF	RoosterBio^®^	hMSCs	Hogan et al. (2019)
MSC NutriStem^®^ XF Medium	Sartorius AG	hMSCs	Li et al. (2021)
Mesenchymal Stem Cell Medium-ACF (XF)	ScienCell Research Laboratories	hMSCs	ScienCell Research Laboratories (2022)
TeSR™-AOF (XF)	STEMCELL Technologies	hiPSCs	STEMCELL Technologies (2023a)
TeSR™-E8™3D (XF)	STEMCELL Technologies	hiPSCs	STEMCELL Technologies (2023b)
MesenCult™-ACF Plus (XF)	STEMCELL Technologies	hMSCs	Hervy et al. (2014)
Cellartis^®^ DEF-CS™ XF	Takara Bio Inc.	hiPSCs	Abecasis et al. (2017)
Cellartis^®^ MSC XF	Takara Bio Inc.	hMSCs	Li et al. (2020b)
Essential 8™ (XF)	Thermo Fisher Scientific	hiPSCs	Chen et al. (2011)
Essential 8™ Flex (XF)	Thermo Fisher Scientific	hiPSCs	Giacomelli et al. (2020)
StemPro™ MSC SFM	Thermo Fisher Scientific	hMSCs	Hervy et al. (2014)
TransStem™ CD XF Human Pluripotent Stem Cell Medium	TransGen Biotech Co., Ltd.	hiPSCs	TransGen Biotech Co., Ltd. (2022a)
TransStem^®^ SF, XF Human MSC Medium	TransGen Biotech Co., Ltd.	hMSCs	TransGen Biotech Co., Ltd. (2022b)
MSC-GRO™ VitroPlus III SF, XF Medium	Vitro Biopharma	hMSCs	Vitro Biopharma (2023)

*hMSCs* human mesenchymal stem cells, *hiPSCs* human-induced pluripotent stem cells

### Downstream processing

In the context of cell therapeutic production processes, downstream processing has received less attention in peer-reviewed literature than its upstream counterpart. It therefore comes as no surprise that there are still challenges and bottlenecks associated with downstream processing during the production of cell therapeutics. These include the short time window available between cell detachment and cryopreservation before quality becomes unacceptable (Viswanathan and Hematti 2017; Scibona and Morbidelli 2019) and the cells shear sensitivity, which restricts rigorous operations in favor of cell recovery (Cunha et al. 2015a; Scibona and Morbidelli 2019). In this context, the first step is the detachment of the hMSCs or hiPSCs from their growth surface as well as from each other. This is usually achieved by adding a proteolytic agent, such as TrypLE or Accutase, to cleave the integrin necessary for cell-to-surface/scaffold attachment, and chelating agents, such as Versene, to scavenge divalent ions required for cell-to-cell junctions (Derakhti et al. 2019). Alternatively, dissolvable scaffolds may be enzymatically digested instead (Rodrigues et al. 2019), or a temperature shift performed to affect cell release if a thermoresponsive coating was used (Narumi et al. 2020). For microcarrier or spheroid-based cultivations, cell recovery rates during detachment may further be improved by increasing fluid dynamic stress and collisions within the system (Nienow 2021), as has been demonstrated in various SU bioreactors with *Φ* between 10 and 25 at impeller speeds of 2 − 5 ∙ *N*_*s*1_, corresponding to a *ϵ*_*max*_ of 1310–2830 mW kg^−1^ and a *λ*_*c*_ of 24–30 μm (Nienow et al. 2016a; Nienow et al. 2016b).

Following complete detachment from carriers, the cells must be separated from any non-dissolvable debris (Viswanathan and Hematti 2017). This so-called clarification is generally realized through dead-end filtration using, for example, SU products such as the Thermo Scientifics’ Harvestainer™ BioProcess Container (Jossen et al. 2018), Entegris’ Microcarrier and Cell Separation System (Pandey et al. 2020), or Merck’s OptiCap^®^ capsules (Cunha et al. 2015a). Various studies have shown that pore diameters >75 μm resulted in cell recovery rates of >80 %, while ensuring a high cell quality and efficient microcarrier removal (Schirmaier et al. 2014; Cunha et al. 2015b; Serra et al. 2018).

After clarification cells are concentrated and then washed by diafiltration. Due to time constraints, both of these processes are often integrated (Cunha et al. 2015a; Pandey et al. 2020), e.g., by tangential flow filtration (TFF), using either hollow-fiber modules or flat sheet cassettes (Viswanathan and Hematti 2017; Cunha et al. 2017). Studies with TFFs highlighted that cell recovery rates and protein clearance improved when processing was continuous (Cunha et al. 2015a). Protein clearance could further be improved by adding a negative mode expanded bed adsorption step prior to concentration and washing (Cunha et al. 2016), while cell recovery rates were shown to be dependent on the choice of system, material, and operating parameters (Cunha et al. 2015b; Cunha et al. 2017). SU TFF technologies used for the downstream processing of hMSCs (Cunha et al. 2017) include Asahi Kasei’s BioOptimal™ MF-SL Microfilters (Cunha et al. 2015a), Cytiva’s ReadyToProcess™ HF microfiltration cartridges, Merck’s Pellicon^®^ XL Cassettes, and Sartorius’ Sartocon^®^Slice 200 (Cunha et al. 2017).

An alternative scalable low shear approach capable of integrating cell concentration and washing is continuous centrifugation (counterflow and disk stack centrifugation). While counterflow centrifugation has been used to purify hMSCs and hiPSCs at different scales (Li et al. 2019; Li et al. 2019; Pandey et al. 2020; Li et al. 2020a), there have been no reports using disk stack centrifugation. A design-of-experiment study performed with a counterflow centrifuge (CTS Rotea) showed that, while neither flowrate, centrifugal force-to-flowrate ratio, serum concentration, nor trypsin concentration impacted live hMSC recovery during centrifugation, the first two did negatively influence cell metabolism (Li et al. 2020a). Further studies showed that these systems not only shorten the process time (Li et al. 2019), but also allow the selective isolation of cell populations based on physical properties, such as size, density, and even viability (Li et al. 2022). Furthermore, the scalability of these systems was demonstrated in a recent study where 3 L cell suspensions with a density of 0.9–3.4 × 10^6^ hiPSCs mL^−1^ were processed within 30 min, achieving a 105-fold concentration and cell recovery rates of up to 99 %, while maintaining target cell quality. The authors did, however, highlight that the chamber capacity would present the main scale-up constraint when moving to 50 L production (Pandey et al. 2020). Commercially available SU continuous centrifugation systems used for the downstream processing of hMSCs and hiPSCs include Sartorius’ kSep^®^ (Pandey et al. 2020), Thermo Scientific’s CTS Rotea Counterflow Centrifuge (Li et al. 2020a), and Terumo BCT’s Elutra Cell Separation System (Li et al. 2022). After this downstream operation, cells are formulated in preparation for cryogenic storage, filled into vials or bags, visually inspected, and frozen away for transport to the site of administration. A more detailed description of these operations is given elsewhere (Viswanathan and Hematti 2017).

## Conclusions and outlook

Allogeneic hMSC- and hiPSC-based therapeutics are on the rise, with a clear trend towards their production in stirred SU bioreactors on microcarriers. Nevertheless, securing clinically relevant cell quantities and quality continues to pose a challenge. In upstream processing, safety issues surrounding hiPSCs need to be addressed and SU perfusion systems adapted to deal with cell shear sensitivity and bioreactor scale. Moreover, suitable chemically defined expansion and differentiation media compositions remain to be developed and commercialized. Here medium compatibility with perfusion mode could be advantageous. In the meantime, the groundbreaking work already done with hMSCs and microcarriers may be adapted to hiPSCs to increase process scale and yield using SU bioreactors, while bearing in mind the inherent similarities and differences between the two cell types.

Ensuring a high cell yield during the cultivation process is only the first step, however. Cells must still undergo downstream operations to meet the quality and purity standards set by regulatory bodies. This demands the development of scalable automated systems capable of time sensitive cell processing. SU downstream processing technologies which combine process steps, such as TFF and continuous centrifugation have proven themselves in this regard, yet more research is necessary to fully understand how these systems impact cell quality and recovery. Finally, new technologies with great developmental potential, such as SU acoustic wave separators (Merck’s ekko™) and SU disk stack centrifuges (GEAs kytero and Alfa Lavals CultureOne), have only recently become commercially available and remain to be tested for stem cell application.
